# Pattern and pace of morphological change due to variable human impact: the case of Japanese macaques

**DOI:** 10.1007/s10329-021-00933-7

**Published:** 2021-08-17

**Authors:** Madeleine Geiger

**Affiliations:** Palaeontological Institute and Museum, University of Zurich, Karl-Schmid-Strasse 4, CH-8006 Zurich, Switzerland

**Keywords:** *Macaca fuscata*, Rate of change, Rate of evolution, Wild, Captive, Anthropophily

## Abstract

Human impact influences morphological variation in animals, as documented in many captive and domestic animal populations. However, there are different levels of human impact, and their influence on the pattern and rate of morphological variation remains unclear. This study contributes to the ongoing debate via the examination of cranial and mandibular shape and size variation and pace of change in Japanese macaques (*Macaca fuscata*). This species is ideal for tackling such questions because different wild, wild-provisioned, and captive populations have been monitored and collected over seven decades. Linear measurements were taken on 70 skulls from five populations, grouped into three ‘human impact groups’ (wild, wild-provisioned, and captive). This made it possible to investigate the pattern and pace of skull form changes among the human impact groups as well as over time within the populations. It was found that the overall skull shape tends to differ among the human impact groups, with captive macaques having relatively longer rostra than wild ones. Whether these differences are a result of geographic variation or variable human impact, related to nutritional supply and mechanical properties of the diet, is unclear. However, this pattern of directed changes did not seem to hold when the single captive populations were examined in detail. Although environmental conditions have probably been similar for the two examined captive populations (same captive locality), skull shape changes over the first generations in captivity were mostly different. This varying pattern, together with a consistent decrease in body size in the captive populations over generations, points to genetic drift playing a role in shaping skull shape and body size in captivity. In the captive groups investigated here, the rates of change were found to be high compared to literature records from settings featuring different degrees of human impact in different species, although they still lie in the range of field studies in a natural context. This adds to the view that human impact might not necessarily lead to particularly fast rates of change.

**Supplementary Information:**

The online version contains supplementary material available at 10.1007/s10329-021-00933-7.

## Introduction

The interactions of humans with non-human animals vary in intensity from anthropophily to intensive breeding, and these intensity levels are part of one continuum, making a strict categorisation difficult (Vigne 2011; Zeder 2012). A gross categorisation can be described as outlined in Table 1, with some grades being nested within the definition of others. Along this gradient of human–animal interaction, morphological changes have been found to occur and have been assumed to be due to human proximity (e.g., Herre and Röhrs 1990; O'Regan and Kitchener 2005; Szulkin et al. 2020). For example, red fox (*Vulpes vulpes*) populations inhabiting urban London have been found to exhibit shorter and broader muzzles compared to their rural conspecifics (Parsons et al. 2020); skull shape variation in lions (*Panthera leo*) and tigers (*P. tigris*) has been found to be influenced nearly twice as much by the captivity status of these animals than by their sex (Hartstone-Rose et al. 2014). Domestication and breed formation is also associated with morphological changes, including, among others, a shortening of the rostrum and a decrease in brain size (e.g., Herre and Röhrs 1990).

**Table 1:** Intensities of human–animal relationships

Grade of intensity of human–animal interaction	Description
Anthropophily (sensu lato) (e.g., urban red foxes and blackbirds in Europe)	Wild animals attracted to human environments and activities
Commensalism (sensu lato) (e.g., black rats in Northern Europe)	Special case of anthropophily Living within human buildings
Captivity (e.g., animals in zoos, working elephants from India and South East Asia)	Humans maintaining wild animals in captivity Not necessarily including human-controlled reproduction in captivity
Domestication (e.g., domestic dogs and chickens worldwide)	Adaptation into the human-dominated ecological niche, including reproductive change Not necessarily including reproduction being controlled by humans
Livestock/pet breeding (e.g., specialised cattle breeds for high-yield meat or milk production)	Special case of the domestication process Deliberate, artificial selection (control of reproduction) for aesthetic or productive traits In itself variable in degree (extensive to intensive) Leading to landrace/breed formation

Human–animal interactions can be described as a gradient, consisting of different, nested grades of increasing intensity (increasing intensity from top to bottom, with some grades being nested within others) (Vigne 2011; Zeder 2012). Descriptions are partially based on Hulme-Beaman et al. (2016) and Sánchez-Villagra (2021)

Within the set of morphological changes occurring because of human–animal interactions, skull shape and size are particularly relevant due to their importance for the individual’s survival. This is because craniofacial morphology is crucial for energy intake (jaws and teeth), as well as sensory (bony structures of the oronasal, ocular, and auricular systems) and cognitive (braincase) capacity. Understanding the variation in these bony structures in response to different degrees of animal–human interaction (Table 1) in different environments yields insights into possible adaptive, plastic, or even random processes that might be or become relevant in the human-dominated ecological niche. Such insights are not only relevant for a better understanding of long-term evolutionary processes in deep time, but might also become relevant for understanding potential phenotypic responses and adaptations as a consequence of the ever-increasing human influence on ecosystems worldwide.

Beyond the morphological features themselves, human influence may also impact the pace/rate at which these changes occur. Traditionally, 'natural' populations (i.e., not disturbed by humans) are thought to have a relatively slow rate of evolution compared to the pace of change occurring in human-dominated environments, especially in domestication, due to vastly altered environmental conditions and artificial selection (Darwin 1859; Haldane 1949; Drake and Klingenberg 2008). This view is supported by higher estimates of the rate of change/evolution in populations influenced by human activity compared to populations that are less influenced in such a way (Hendry et al. 2008). On the other hand, compared to rates in the ‘wild’, faster, equal, and slower rates of change have all been observed in domestication and breed formation (Purugganan and Fuller 2011; Geiger and Sánchez-Villagra 2018). Similarly, populations in field versus experimental conditions were found to change at a similar pace (Gingerich 2019). Thus, it is evident that the traditional view (slow rates of change/evolution in ‘nature’—fast rates of change/evolution in anthropogenic environments) is challenged, and new data on different taxa and on different grades of intensity of the human–animal relationship are needed to improve our understanding of the tempo of evolution (Gingerich 2019).

In this study, morphological change and the pace at which it is occurring were investigated in the Japanese macaque (*Macaca fuscata*) as a case study. Different populations of these macaques, which are living under different degrees of human impact (i.e., different human impact groups), have been monitored in long-term field study sites since about the mid-twentieth century (Nakagawa et al. 2010; Yamagiwa 2010), thus allowing for intraspecific morphological comparisons among populations and human impact groups. In general, wild Japanese macaques can be classified as an anthropophilic species (Table 1), which regularly habituates to humans and exploits human resources (Suzuki and Muroyama 2010). However, some populations are living in the wild, for example, on small islands with no or only occasional human contact and food provisioning (here classified as ‘wild’). Other populations are free-ranging and associated with monkey parks, where they are regularly provisioned with food (here classified as ‘wild-provisioned’), and some populations are housed in enclosures related to research institutions or zoos (here classified as ‘captive’). Further, some individuals of some populations have been transferred from a wild or wild-provisioned to a captive environment at some point during their more recent history. These human impact groups mirror some of the steps along a gradient from wild to domestic as outlined in Table 1. Osteological material from many of these Japanese macaque populations has been sampled through decades and documented meticulously in institutional and museum collections. Thus, these Japanese macaque populations present an ideal case for studying variation in cranial and mandibular shape and size as well as the pace of this change as a response to different levels of human–animal interactions, despite a recent meta-analysis finding only minor morphological changes in primates due to captivity (Siciliano-Martina et al. 2021).

Japanese macaques are endemic to Japan, where they inhabit warm-temperate evergreen broadleaf forests in the south and cool-temperate deciduous broadleaf forests in the north of their distribution (Abe 2005; Nakagawa et al. 2010). Males are larger than females, with the latter reaching a body mass of about 8–16 kg (Abe 2005). The wide latitudinal distribution of these macaques has been found to be associated with body mass and morphological variation according to Bergman’s rule, with northern populations being larger than southern ones (Hamada and Yamamoto 2010). Accordingly, Koshima macaques in the extreme south are the smallest Japanese macaques, although the insular effect might also play a role (Hamada and Yamamoto 2010). Further, Japanese macaques are omnivorous and semi-terrestrial, walking and running mainly quadrupedally (Chatani 2003; Abe 2005). Females generally stay in their native troop, where they reach sexual maturity at the age of 5–7 years, reproducing about once in 2–3 years, with a gestation period of 173 days (Abe 2005).

In this study, cranial and mandibular dimensions and changes thereof through time were investigated in Japanese macaque populations under variable human impact (wild, wild-provisioned, and captive). First, it was hypothesised that cranial and mandibular dimensions in Japanese macaques from wild, wild-provisioned, and captive macaques would differ from one another due to an increasing degree of human impact. Second, it was hypothesised that potential changes in cranial and mandibular dimensions would be occurring faster the higher the degree of human impact.

## Materials and methods

### Specimens, populations, and human impact groups

**Fig. 1:**
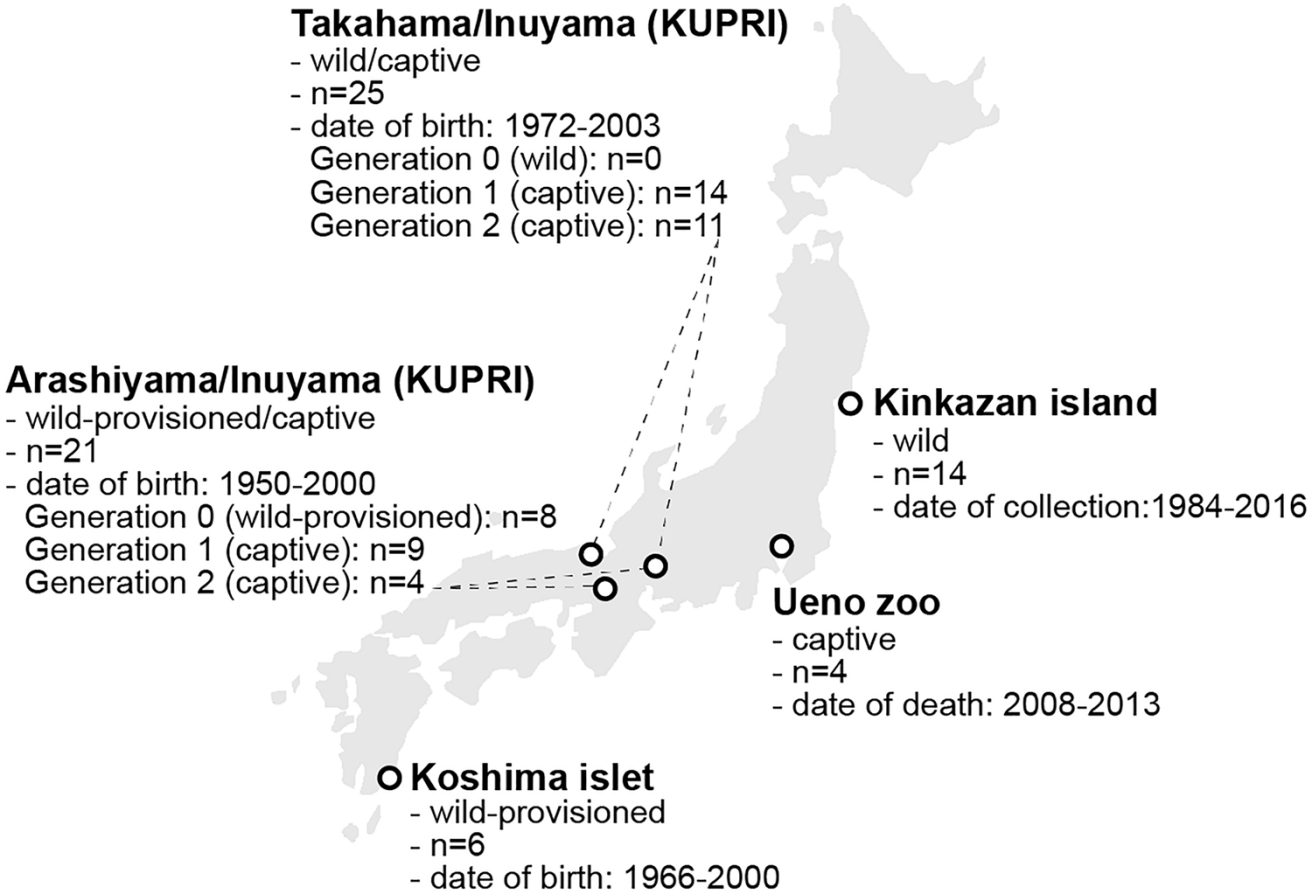
Japanese macaque populations sampled for this study. Approximate geographic position of the populations are shown on a simplified map of Japan. For each of the five sampled populations, the human impact group (wild, wild-provisioned, captive; Nakagawa et al. 2010), the total number of specimens studied (*n*; note that for some analyses the sample size was reduced, see text), the years of birth/death/collection covered, and the number and sample size of studied generations (if known) are given. KUPRI = Primate Research Institute of the Kyoto University. Source of map: https://commons.wikimedia.org/wiki/File:JapanGrey.png

For this study, 70 dry skulls of Japanese macaques (*Macaca fuscata fuscata*) were investigated, originally stemming from five different populations in Japan (Arashiyama, Kinkazan island, Koshima islet, Takahama, and Ueno Zoo) and different ‘human impact groups’ (Fig. 1): ‘wild’ (*n* = 14), free-ranging, not generally provisioned with food; ‘wild-provisioned’ (*n* = 14), free-ranging, generally provisioned with food; ‘captive’ (*n* = 42), not free-ranging, provisioned with food. Specimens cover a range of years of birth/death (1950–2016) and generations (0–2) (Fig. 1). In 1970 and 1981, individuals from the wild Takahama and the wild-provisioned Arashiyama populations, respectively, were transferred to outdoor enclosures at the Kyoto University Primate Research Institute in Inuyama (Huffman 1991; Nahallage and Huffmann 2007) (Fig. 1). In these two groups, the founders (wild-provisioned: Arashiyama; wild: Takahama) have been defined as generation 0 in the collection database (Fig. 1). The offspring from generation 0, which have been raised in captivity (here categorised as captive) in both the Arashiyama and the Takahama groups, have been defined as generation 1, and the subsequent offspring as generation 2 (Fig. 1). Note that although the Arashiyama and the Takahama specimens used in this study thus comprise a subset of their respective populations in the wild, the term ‘population’ in connection with these groups is still used in the remainder of this paper to prevent confusion with the human impact groups. In the Ueno Zoo, where the northernmost population of macaques of the Shimokita Peninsula is being kept, the specimens are breeding in the zoo, with only occasional introductions of males from the wild to maintain genetic diversity (Aoki et al. 2015). Skulls are housed at the Primate Research Institute of the Kyoto University, Inuyama (KUPRI), and the National Museum of Nature and Science, Tokyo (NSMT).

Due to a pronounced sexual dimorphism, female philopatry, and in many cases, greater numbers of females in the collections, only skulls from females were used for this study. Further, only skulls of dentally and skeletally mature specimens were used in order to exclude the effect of ontogenetic variation. Dental maturity was here defined to be attained if all permanent teeth had erupted into occlusion (Kamaluddin et al. 2019). Skeletal maturity was defined to be attained if the growth plates of the humerus and femur (if available) were at least partially fused in at least one of the two sides of the body (left or right).

The age at death is known for 50 of the investigated specimens from the Arashiyama, Koshima (not all), and Takahama populations. The age range of these specimens is 6.9–30.4 years, with a mean of 15.4 years and a standard deviation of 5.9 years. Although the age at adulthood, i.e., the age when growth of the body ceases, has not yet been determined in Japanese macaques, the age at dental maturity, i.e., the eruption of the third molars, which has been found to be at about 7 years of age, is generally regarded as the age demarcating adulthood (Ideka and Watanabe 1966; Hamada et al. 1986, 1996; Iwamoto et al. 1987; Hamada and Yamamoto 2010; Kamaluddin et al. 2019). This age at adulthood corresponds to the minimal age of specimens studied here. However, body size and skull shape, especially facial dimensions, in Japanese macaques change not only during juvenile growth (which was excluded here), but also during adulthood with increasing age (Hamada and Yamamoto 2010; Kamaluddin et al. 2019). The effect of age was evaluated in the analyses (see below).

Information on birth and death dates, number of generations in the respective human impact group (e.g., in captivity), and relatedness of individuals was collected from collection labels or collection databases. Some of the specimens from Arashiyama and Takahama are known to be related to each other (mother and offspring/siblings). Generally, only specimens with a known year of birth were used so that the rate of morphological change could be evaluated (see below). However, in some populations the year of birth was not known. Notably, in the zoo population (Ueno), only the death date was known, and in one wild population (Kinkazan) only the collection date was known (Fig. 1; potential drawbacks of the use of death/collection dates are discussed below). Specimens were deliberately chosen to represent the widest possible range of birth years (or year of death/year of collection), in order to investigate potential trends of change in their skull morphology. Generally, every year for which there was a suitable specimen available (dentally mature, not damaged) that was born/died in that year, a sample was taken. If there was more than one specimen present representing a given year, one specimen was randomly chosen in order to not bias the data set in favour of years for which there were many specimens available (and to save time).

In one population (Kinkazan island), the date of birth and the date of death is not known. In these, the collection date was used as the date of death (Fig. 1). In the wild populations, individuals die of natural causes, and if their carcases are found, they are entered into the collection for study (Takeshi Nishimura pers. comm.). The collection date may be different from the date of death, because it might take a while before a deceased individual is noticed and recovered. However, care was taken to not sample specimens that showed obvious signs of having died long (years) before they were found, i.e., specimens having remains of plant particles in their cranial cavities. Therefore, the year in which these specimens were found is probably close to the year in which they were collected.

### Measurements

**Fig. 2:**
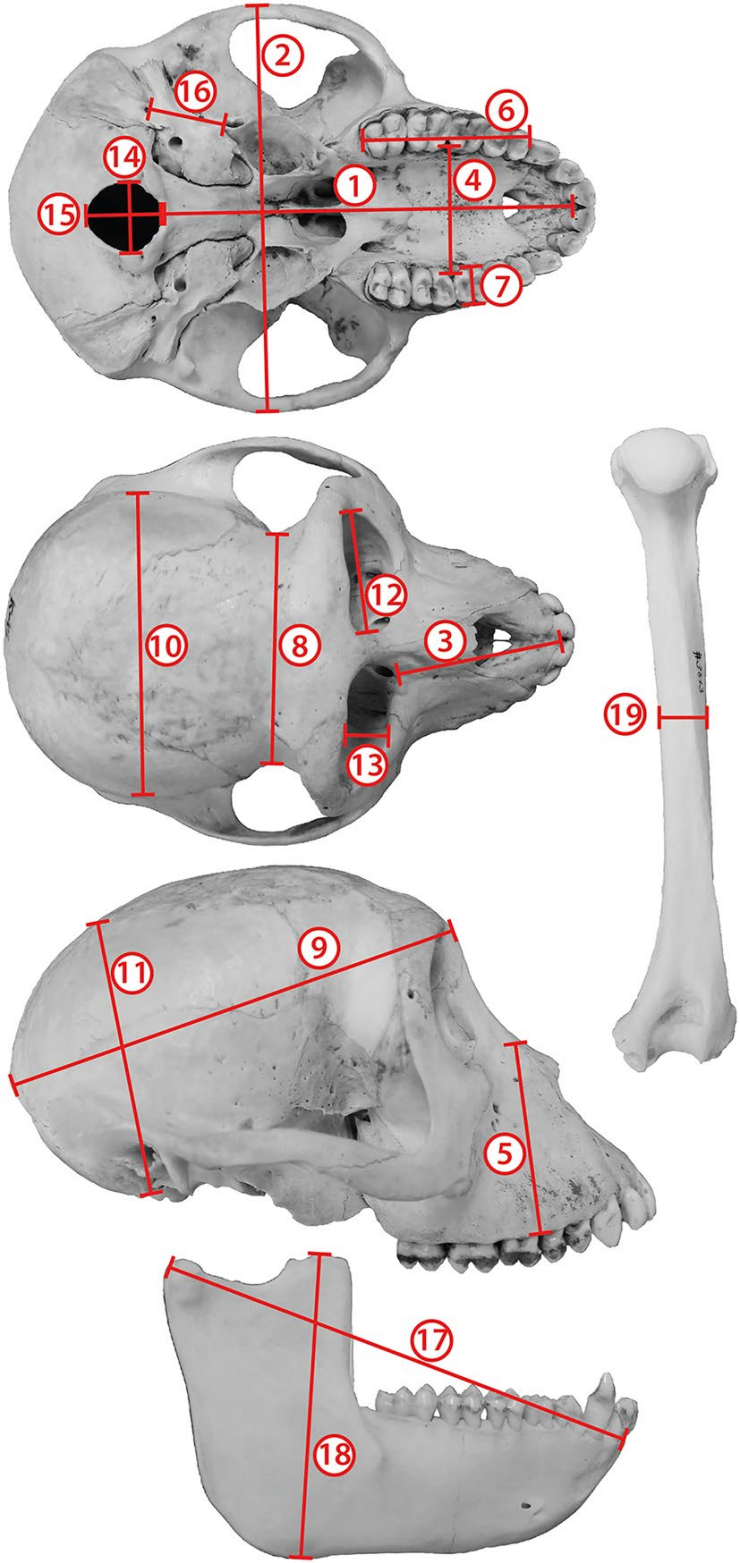
Measurements taken in this study. Numbers correspond to the detailed descriptions in Table 2. Cranium is specimen KUPRI # 8896, humerus and mandible are specimen KUPRI # 3013. KUPRI = Primate Research Institute of the Kyoto University

Eighteen measurements were taken on the cranium and the mandible, as described in Table 2 and Fig. 2, with a digital calliper to the nearest 0.01 mm. Measurements were chosen so that they reflect the shape of the different main parts of the skull (e.g., length, height, and breadth of the braincase and the rostrum) as well as functionally important structures [e.g., postcanine toothrow length, breadth of the first upper molar (M1)]. Unilateral measurements were taken on the left side of the cranium and the mandible, unless damage or diseased tissue on the left made necessary taking measurements on the right side. Due to missing, diseased, or worn teeth, broken bone parts, and/or post-mortem cutting of the cranial vault, not all measurements could be taken in all specimens.

**Table 2:** Measurements of the cranium, mandible, and humerus used in this study

No.	Measurement	Description
1	Cranial length	Most anterior point of the premaxilla, at the level of the alveoli of the first incisors in ventral view (alveolare), to the rostral border of the foramen magnum (basion)
2	Cranial breadth	Maximum breadth of the zygomatic arches, perpendicular to the long axis of the cranium
3	Length of rostrum	Most anterior point of the premaxilla at the level of the alveoli of the first incisors in dorsal view (prosthion) to the most anteromedial edge of the orbit
4	Palatal breadth	Maximum breadth of the palate, measured at the internal margins of the left and right upper tooth rows between M1 and M2 at the level of the alveoli
5	Height of rostrum	Vertical height of the rostrum, measured from the intersection of nasal, premaxilla, and maxilla to the alveolar edge between P4 and M1
6	Length of postcanine tooth row	Length of the tooth row posterior to the canine teeth (including two premolars and three molars), measured at the level of the alveoli and on the buccal side of the tooth row
7	Breadth of M1	Maximum buccolingual width of the upper first molar, measured at the enamel-dentin junction
8	Postorbital constriction breadth	Minimum breadth of the narrowing of the cranium behind the orbits
9	Braincase length	Most protruding point of the frontals between the eyes (at about the position of the nasofrontal sutures) to the most posteriorly protruding point of the external occipital protuberance
10	Braincase width	Widest part of the parietal and/or squamosal (temporal) bones, right above the external auditory meatus
11	Braincase height	From the level of the basioccipital bone to the highest point on the top of the cranial vault, excluding the sagittal crest if present, and approximately where the frontoparietal and interparietal sutures meet
12	Orbital width	Maximum mediolateral extension of the orbit, measured at the fronto-zygomatic suture on the lateral side and the maxilofrontal suture on the medial side
13	Orbital height	Maximum dorsoventral extension of the orbit, parallel to the medial line of the face
14	Foramen magnum width	Maximum mediolateral extension of the foramen magnum
15	Foramen magnum height	Maximum dorsoventral extension of the foramen magnum
16	Interforaminal distance	Distance between posterior edge of foramen ovale and anterior edge of stylomastoid foramen
17	Mandibular length	Distance between the tip of the mandible, at the level of the alveoli of the first incisors, to the most distal point of the condylar process
18	Mandibular height	Vertical distance between the highest point of the coronoid process to the base of the ascending ramus of the mandible
19	Humerus shaft diameter	Mediolateral diameter of the shaft of the humerus in posterior view, measured directly distal to the deltoid tuberosity and in a right angle to the long axis of the bone

Measurements are according to Snell-Rood and Wick (2013) and Geiger and Sánchez-Villagra (2018), or developed for the present study. For depictions see Fig. 2

In addition to the cranial and mandibular dimensions, the diameter of the right humerus (Table 2) was measured as a proxy for body size in each specimen. The stylopodal skeletal elements, including the humerus in mainly quadrupedal animals such as the Japanese macaque, serve to provide suitable estimates of body mass in mammals because of their weight-bearing properties (Gingerich 1990; Jungers 1990). All raw measurements used in this study can be obtained from Online Resource 1.

The first ten specimens (replication test specimens) measured for the current study were measured twice: once in the beginning of the measurement period and a second time 1 day later. These replications made it possible to evaluate whether the measurements were replicable and whether the measurement procedure was kept constant. If replicates were so different as to assume a fault in either one or the other measurement, both replicates were repeated, and the measurement procedure was adjusted to be more replicable for all specimens. For every measurement, the standard deviation of the two replicates in each of the ten replication test specimens was then calculated. The mean of these ten standard deviations was subsequently compared to the mean standard deviation across all specimens of that population for that measurement. This procedure was repeated for all measurements and allowed for an evaluation of measurement error, i.e., error regarding the replicability of a measurement, with the actual (biological) variation among specimens. This evaluation of replicated measurements showed that the standard deviation between the replicates, which are errors implied by the observer (M.G.), were on average 14.1 times lower (minimum 1.93 times, maximum 76.94 times) than the standard deviation among the specimens. It was thus assumed that the repeatability of these measurements was sufficient for further analyses.

### Allometry adjustment of measurements

Allometry adjustment was conducted to account for potential size differences among the populations as a result of climatic adaptations (e.g., Bergman’s rule; Hamada and Yamamoto 2010) as well as for the effects of inter-individual variation in body size, which may influence skull shape (allometric scaling). Prior to further analyses, all measurements were log10 transformed. As a proxy for body mass, humerus diameter was used (see above). To adjust the cranial and mandibular measurements for allometric scaling, the log10-transformed cranial and mandibular measurements were regressed against the log10-transformed humerus diameter. The residuals of these regressions were subsequently extracted as allometry-adjusted measurements to use for further statistical analyses.

### Analyses of overall skull shape and size variation in the human impact groups

To determine whether the overall cranial and mandibular shape and size of the different Japanese macaque populations under variable human influence varied according to their human impact group (wild, wild-provisioned, captive), principal components analyses (PCA) based on a covariance matrix were conducted. For PCA, several cranial dimensions represented by missing measurements in many specimens had to be excluded (these included braincase length, braincase width, braincase height, and breadth of M1). Further, 14 specimens with missing single measurements were excluded. In sum, the PCA was conducted using 14 log10-transformed cranial and mandibular measurements and 56 specimens from all populations. PCA was repeated using allometry-adjusted measurements. Linear regressions were used to deduce how strongly body size (i.e., humerus diameter) and individual age at death correlate with PC1 and allometry-adjusted PC1 across all specimens. Differences in PC1 and allometry-adjusted PC1 among human impact groups were examined using non-parametric Kruskal–Wallis tests for equal medians and post hoc Mann–Whitney pairwise comparisons due to small sample sizes within human impact groups.

The PC1 scores (non-allometry-adjusted and allometry-adjusted) were subsequently used to calculate whether cranial and mandibular shape were correlated with the year of birth (or year of collection, if the year of birth was not available in certain populations, Fig. 1) within each population. For this, Spearman’s correlations were used, due to relatively small within-population sample sizes for some populations. In cases of an at least medium effect correlation coefficient (*r* ≥ 0.3) as well as low significance values (*p* ≤ 0.1) between year of birth/collection and PC1 in a population, it was deduced that there was evidence for a change in skull shape over time in that population. The Ueno Zoo population was not considered in this analysis because only one specimen had a sufficiently complete set of measurements to be included in the PCA.

### Analysis of skull shape variation and rate of change in two selected Japanese macaque populations

The year of birth and, in particular, the year of death are not optimal proxies to characterise change over time within a population in a species with relatively long generations, such as the Japanese macaque. This is because sexual maturity occurs relatively late (Abe 2005) and generations overlap, so that a different year of birth does not imply a different generation. Furthermore, the relatively long lifespan of this species renders the linkage between year of death and generation potentially weak. Thus, two of the investigated populations, in which the generation of every specimen was known (Arashiyama and Takahama, Fig. 1), were analysed in more detail than the other populations. For these analyses, the full set of specimens and measurements was used.

#### Arashiyama (wild-provisioned/captive)

In this population, the female members of three consecutive generations could be measured. Generation 0 comprises individuals that were captured from the original wild-provisioned population. Generations 1 and 2 comprise individuals from consecutive generations in captivity (Fig. 1). To discern whether there were differences among generations concerning different cranial and mandibular measurements, Kruskal–Wallis tests were performed, with cranial and mandibular measurements as the dependent variables and the three generations as independent variable. The same analysis was performed for non-allometry-adjusted and allometry-adjusted measurements. As body mass (in kg) at death was available for 19 specimens and age at death was available for all 21 specimens from the collection database, variation in body mass and age at death over generations was also analysed.

#### Takahama (wild/captive)

In this population, the female members of two consecutive generations could be measured. Generation 0, which comprises individuals that were captured from the original wild population, could not be measured, but generation 1 and 2 comprise individuals from consecutive generations in captivity (Fig. 1). Also, here, due to the small and unequal sample size within these generations, non-parametric pairwise Wilcoxon tests to compare the medians of the measurements between generations were computed. Apart from the different test statistics, all analyses were conducted as described above for the Arashiyama population. Body mass at death (in kg) was available for 22 specimens and age at death for all 25 specimens from the collection database.

#### Estimation of rate of change

 For both of these populations (Arashiyama and Takahama), the rate of change was analysed as a next step, using those allometry-adjusted measurements which were found to show some degree of difference between generations (if *p* < 0.1). Allometry-adjusted data were used because size and age may influence cranial and mandibular dimensions. Calculations were made between generations 0 and 1 (if available) and generations 1 and 2 in the Arashiyama and Takahama populations. In other words, generation length was equal to one for all comparisons (step rates). For calculating the rates of change, Haldane estimates (standard deviation per generation) were used: [(x2 − x1)/sd]/[t2 − t1], where x1 and x2 are the sample means of residual log10-transformed measurements (i.e., allometry-adjusted measurements) in two subsequent generations, and sd is the pooled standard deviation of x1 and x2 across the time points (Gingerich 1993; Anderson and Handley 2002; Purugganan and Fuller 2011). As in the current study the number of elapsed generations between x1 and x2 equals 1, no division by the number of elapsed generations was performed. Note that usually, instead of log10 transformation, ln transformation is used to calculate Haldanes. However, because the log10-transformed measurements were divided by the standard deviation of log10-transformed measurements, variation in data transformation methods did not influence the results.

These rate estimates were subsequently compared to estimates provided in a recent literature review that also compared step rates in populations under variable human impact. For this, step rate estimates from experimental selection studies and field studies were extracted from an extensive literature review published by Gingerich (2019). From these detailed per-trait records, species mean step rates per reviewed study were extracted. For experimental selection studies, only step rates not resulting from control experiments were included, to reflect artificial selection pressure. Field studies were categorised into two different groups according to Hendry et al. (2008): ‘anthropogenic context’, including in situ anthropogenic disturbance (e.g., urbanisation, taming), introduction of a population into a new habitat (comparison of introduced with ancestral populations), or introduction of a new host into the range of a native species; and ‘natural context’, including in situ natural variation without obvious human impact, self-induced range or host expansion, or range expansion after introduction, with populations spreading on their own after being introduced by humans. According to the traditional view put forward above, the assumption would be that step rate estimates from experimental selection studies are larger than the ones from field studies, whereas step rates from field studies in an anthropogenic context are larger than those from a natural context. Subsequently, single-case tests were computed using the mean step rate of the Arashiyama and the Takahama population as the single case to test whether it was similar to step rate estimates in field studies in a natural context, field studies in an anthropogenic context, or the experimental selection studies gathered by Gingerich (2019). Due to the influence of body size on measurements, rate of change in humerus shaft diameter as a proxy for body size was analysed as well. All analyses were conducted using Microsoft Excel 2013, Past (version 3.15), and R (version 4.0.2).

## Results

### Cranial and mandibular form variation among Japanese macaque human impact groups

**Fig. 3:**
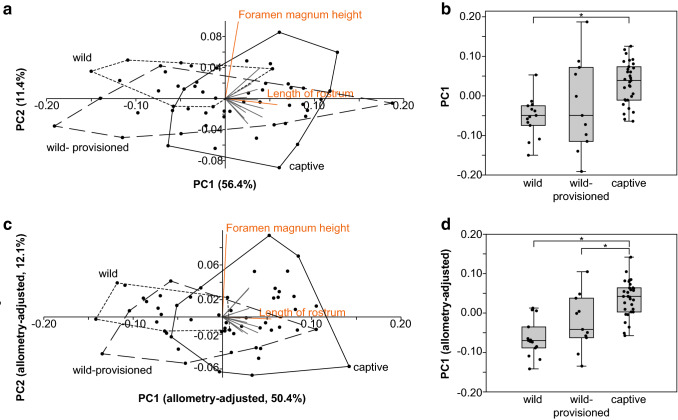
Comparison of cranial and mandibular shape in different human impact groups of Japanese macaques. Principal component analysis of non-allometry-adjusted (**a**) and allometry-adjusted (**c**) cranial and mandibular measurements. Black convex hulls indicate the shape space of the different human impact groups (see text, Fig. 1). Grey straight lines indicate contributions of the different measurements to principal components (PC) 1 and 2, with longer lines indicating greater influence in the direction of the line. Measurements contributing the most for each PC are indicated in orange. (For more detailed information on the loadings see Online Resource 2, Fig. S1.) Box plots (with jittered points) indicate the distribution of PC1 scores based on non-allometry-adjusted (**b**) and allometry-adjusted (**d**) measurements among the human impact groups. Asterisks indicate the groups that are significantly different from each other on a 5% level

PCA with non-allometry-adjusted log10-transformed skull measurements resulted in the first two principal components explaining 67.8% of the total variance, with the first principal component (PC1) explaining 56.4% and the second principal component (PC2) explaining 11.4% of the variation in the data (Fig. 3a). PC1 was moderately and significantly correlated with the body size proxy used (humerus length; *R*^2^ = 0.26, *p* < 0.0001) and with individual age at death (*R*^2^ = 0.14, *p* = 0.018). PCA on allometry-adjusted data resulted in the first two principal components explaining 62.5% of the total variance, with PC1 explaining 50.4% and PC2 explaining 12.1% of the variation in the data (Fig. 3c). The allometry-adjusted PC1 was not correlated with individual age at death (*R*^2^ = 0.028, *p* = 0.30). It could therefore be assumed that correction for size also corrected for age-related variation in the data.

In both non-allometry-adjusted and allometry-adjusted data, the measurement entering most strongly into PC1 was rostrum length, and the measurement entering most strongly into PC2 was foramen magnum height (Fig. 3a, c; Online Resource 2, Fig. S1). There was substantial overlap among the human impact groups (Fig. 3a, c). However, along non-allometry-adjusted and allometry-adjusted PC1, the wild group clustered towards negative PC1 scores, while the captive group was clustered more towards positive PC1 scores, with the wild-provisioned group occupying the entire shape space (Fig. 3a, c). There was no discernible segregation of human impact groups along PC2 (Fig. 3a, c).

The comparison of PC1 scores among the three human impact groups revealed significant differences for non-allometry-adjusted PC1 (*H* = 15.59, *p* < 0.001; Fig. 3b) and allometry-adjusted PC1 (*H* = 23.17, *p* < 0.001; Fig. 3d). In both comparisons, the wild group was significantly different from the captive group (non-allometry-adjusted, *U* = 49, *p* < 0.001; allometry-adjusted, *U* = 29, *p* < 0.001), while in the allometry-adjusted comparison the captive and the wild-provisioned groups were also different (*U* = 78, *p* = 0.008) (Fig. 3b, d). This again showed that rostrum length, which is the variable contributing most heavily to PC1 in both non-allometry-adjusted and allometry-adjusted analyses, was longer in the investigated captive macaques relative to the wild ones.

### Variation in cranial and mandibular form over time

The results of correlations of non-allometry-adjusted PC1 and allometry-adjusted PC1 scores on year of birth/collection as a proxy for time are shown in Table 3. Directed changes over time in skull form appear to have occurred only in the Arashiyama and the Takahama populations (Table 3). In the wild-provisioned/captive Arashiyama population, PC1 and therefore mainly the rostrum became shorter with time (Table 3). However, since no evidence for this correlation regarding the allometry-adjusted PC1 was found (Table 3), these changes were probably primarily related to body size variation. In the wild/captive Takahama population, allometry-adjusted PC1 increased slightly (yet not significantly on a 5% level) over time, which is consistent with a size-independent lengthening of the rostrum over time (Table 3). Morphological changes occurring over generations in the Arashiyama and the Takahama are scrutinised more closely in the following section of the results.

**Table 3:** Results of correlations of non-allometry-adjusted and allometry-adjusted PC1 scores on year or birth/collection

Population, human impact group, number of specimens	Used proxy for time and studied time span	Correlation of non-allometry-adjusted PC1 scores with time	Correlation of allometry-adjusted PC1 scores with time
Arashiyama, wild-provisioned/captive, *n* = 15	Year of birth: 1962–2001	***r* = −0.577; *p* = 0.024**	*r* = **−**0.057; *p* = 0.840
Koshima islet, wild-provisioned, *n* = 6	Year of birth: 1966–2000	*r* = **−**0.265; *p* = 0.633	*r* = **−**0.145; *p* = 0.778
Kinkazan island, wild, *n* = 13	Year of collection: 1984–2016	*r* = 0.389; *p* = 0.189	*r* = **−**0.372; *p* = 0.210
Takahama, wild/captive, *n* = 21	Year of birth: 1972–1999	*r* = 0.055; *p* = 0.812	***r* = 0.387; *p* = 0.082**

Bold font indicates correlations with at least a medium effect correlation coefficient (*r* ≥ 0.3) as well as low significance values (*p* ≤ 0.1); *n* number of specimens

### Differences in skull dimensions and rate of change among generations in the Arashiyama and Takahama populations

**Fig. 4:**
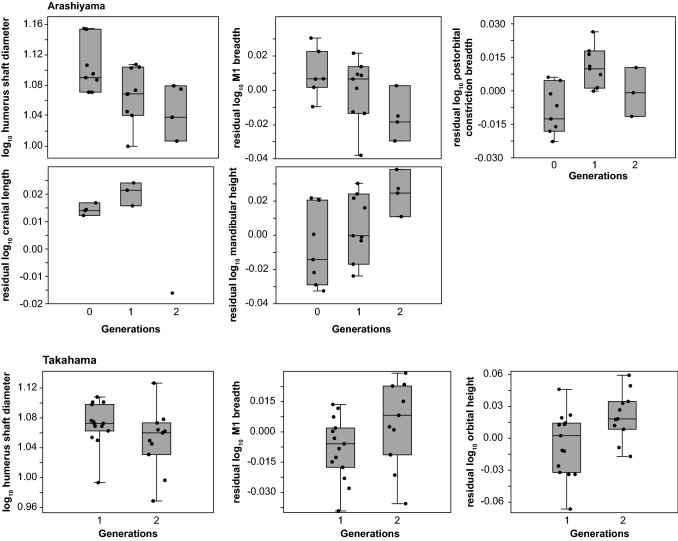
Comparisons of allometry-adjusted cranial and mandibular measurements among generations in the wild-provisioned/captive Arashiyama and the wild/captive Takahama populations. Shown are allometry-adjusted skull measurements that were found to exhibit some degree of difference between the generations (if *p* < 0.1), as well as humerus shaft diameter as a proxy for body mass (Table 4)

Differences among the three generations in the wild-provisioned/captive Arashiyama population were found regarding non-allometry-adjusted cranial length, cranial breadth, breadth of M1, postorbital constriction breadth, orbital width, interforaminal distance, mandibular length, and humerus shaft diameter (Table 4). Allometry-adjusted data revealed differences in breadth of M1, postorbital constriction breadth, braincase length, and mandibular height among the generations (Table 4; Fig. 4). Differences between the two generations in the wild/captive Takahama population were found in the orbital height and humerus shaft diameter in non-allometry-adjusted measurements and breadth of M1 and orbital height in allometry-adjusted measurements (Table 4; Fig. 4). The median age among the generations was found to be overall similar in both the Arashiyama population (Kruskal–Wallis *H* = 4.223, *p* = 0.121) and the Takahama population (Mann–Whitney *U* = 67.5, *p* = 0.622), so ontogenetic variation is probably negligible. Further, in both the Arashiyama and the Takahama populations, measured body mass at death (kg) was not different among the generations (Arashiyama, Kruskal–Wallis *H* = 1.994, *p* = 0.369; Takahama, Mann–Whitney *U* = 60, *p* = 1). Hence, variation in age and/or size, which might influence skull shape, was similar in all investigated generations, thus not influencing observed differences among the generations.

**Table 4:** Differences in medians and rates of change among generations in the wild-provisioned/captive Arashiyama and the wild/captive Takahama populations regarding non-allometry-adjusted and allometry-adjusted measurements

Measurements	Arashiyama					Takahama				
	Non-allometry-adjusted		Allometry-adjusted		Rate of change^a^	Non-allometry-adjusted		Allometry-adjusted		Rate of change^a^
	*H*	*p*	*H*	*p*	*h*	*U*	*p*	*H*	*p*	*h*
Cranial length	7.590	**0.022**	2.266	0.322	Na	84.500	0.702	57.000	0.292	Na
Cranial breath	4.792	**0.091**	0.136	0.934	Na	75.000	0.936	53.000	0.202	Na
Length of rostrum	3.403	0.182	2.949	0.229	Na	105.000	0.132	95.000	0.345	Na
Palatal breadth	1.124	0.570	0.638	0.727	Na	54.000	0.371	51.000	0.285	Na
Height of rostrum	3.998	0.136	4.296	0.117	Na	76.000	0.439	61.000	0.926	Na
Length of postcanine tooth row	1.533	0.465	0.809	0.667	Na	58.000	0.501	52.000	0.312	Na
Breadth of M1	6.007	**0.050**	5.059	**0.080**	−0.591, −0.866	46.500	0.177	41.000	**0.096**	Na, 0.915
Postorbital constriction breadth	5.370	**0.068**	9.394	**0.009**	1.376, −1.108	85.000	0.396	79.000	0.625	Na
Braincase length	2.722	0.256	4.764	**0.092**	1.412, Na	9.000	0.429	3.000	0.429	Na
Braincase width	3.889	0.143	1.833	0.400	Na	9.000	0.429	8.000	0.643	Na
Braincase height	2.333	0.311	2.333	0.311	Na	3.000	0.429	2.000	0.286	Na
Orbital width	5.017	**0.081**	3.542	0.170	Na	56.000	0.267	56.000	0.267	Na
Orbital height	0.439	0.803	0.192	0.909	Na	38.000	**0.035**	37.000	**0.029**	Na, 0.640
Foramen magnum width	0.466	0.792	0.179	0.914	Na	63.500	1.000	64.000	0.975	Na
Foramen magnum height	1.529	0.466	1.876	0.391	Na	75.000	0.477	73.000	0.557	Na
Interforaminal distance	4.726	**0.094**	3.998	0.136	Na	95.500	0.324	86.000	0.648	Na
Mandibular length	5.000	**0.082**	3.520	0.172	Na	85.000	0.687	60.000	0.373	Na
Mandibular height	1.533	0.465	6.188	**0.045**	0.622, 1.010	92.000	0.434	80.000	0.893	Na
Humerus shaft diameter	5.150	**0.076**	Na	Na	−0.939, −0.542	108.000	**0.095**	Na	Na	Na, −0.585

*H* Kruskal–Wallis test statistic, *h* Haldane estimates of rate of change (standard deviation per generation; only calculated for allometry-adjusted data), *Na* not applicable, *p* significance value, *U* Mann–Whitney test statistic. Bold cells indicate significance values < 0.1. Allometry-adjusted comparisons that are indicated in bold are visualised in Fig. 4

^a^Featuring only allometry-adjusted measurements, except humerus shaft diameter; estimates are given from generation 0–1 before the comma and from generation 1–2 after the comma in the Arashiyama population (except for braincase length, where only one specimen could be measured in generation 2, and there is subsequently no rate estimate from generation 1–2) and from generation 1–2 in the Takahama population

Rates of change in humerus shaft diameter (proxy for body size) and those allometry-adjusted measurements that changed markedly among generations (*p* < 0.1) range in absolute values between 0.542 and 1.412 standard deviations per generation in the wild-provisioned/captive Arashiyama population, with a mean of 0.951 and a median of 0.939 standard deviations per generation (Table 4). Note that the sign of the rate (plus or minus) shows whether a measurement tends to get smaller or larger over time, but for the interpretation of the magnitude of change, only absolute values were used (Geiger and Sánchez-Villagra 2018). In the wild/captive Takahama population, rates of change range between 0.585 and 0.915 standard deviations per generation, with a mean of 0.714 and a median of 0.640 standard deviations per generation (Table 4). The median rate of change was similar between the Arashiyama and the Takahama populations (Mann–Whitney *U* = 8, *p* = 0.355). Taken together, the mean step rate of the Arashiyama and the Takahama populations was 0.891 standard deviations per generation. Single-case tests, using the mean step rate of the Arashiyama and the Takahama population as the single case, showed that the mean step rate of the Japanese macaques was significantly different and by trend larger compared to the field studies in a natural context (mean step rate = 0.148 standard deviations per generation, standard deviation = 0.316 standard deviations per generation; single-case test, *t* = 2.331, *p* = 0.024), the field studies in an anthropogenic context (mean step rate = 0.055 standard deviations per generation, standard deviation = 0.048 standard deviations per generation; *t* = 16.804, *p* < 0.001), and the experimental selection sample (mean step rate = 0.035 standard deviations per generation, standard deviation = 0.10 standard deviations per generation; *t* = 8.183, *p* < 0.001) gathered by Gingerich (2019). However, the mean step rate of the Arashiyama and the Takahama populations lie in a similar range as those reported in field studies in a natural setting, ranging from 0.002 to 2.261 standard deviations per generation (Gingerich 2019).

## Discussion

In this study, variation in cranial and mandibular size and shape in Japanese macaque populations and changes thereof through time under different magnitudes of human impact were investigated (human impact groups: wild, wild-provisioned, and captive). It was hypothesised that, first, cranial and mandibular shape would differ among wild, wild-provisioned, and captive macaques due to an increasing degree of human intervention. Second, it was hypothesized that potential changes in skull form are occurring faster in populations that are under greater human influence relative to populations under less human influence, due to the closer proximity of the first to vastly altered environmental conditions than the latter.

Morphological differences seen among populations may in general be due to phenotypic plasticity, genetic changes (selection and/or genetic drift), or both. In the present study, the mechanisms of the observed changes (genetic or plastic) cannot be determined. However, it is likely that morphological changes occurring over one or two generations within one population result from mainly phenotypic plasticity and only to a lesser degree genetic changes, while inter-population differences are probably the result of mainly genetic changes. In any event, both genetic and plastic changes might play a considerable role in the evolution and diversification of groups (Pfennig et al. 2010). Genetic changes in morphology due to variable human impact could occur via three different, potentially intermingled ways (McPhee 2004): first, directional selection, with a trait changing away from the original mean and as a function of the number of generations in captivity, with the variance in the trait remaining the same; second, relaxed selection due to a release from the natural selection pressures in the wild, leading to more variability in a trait, with the mean not necessarily being changed, as a function of the number of generations in captivity; and third, genetic drift due to a small founder population, leading to differences in trait mean and variance without a predictable pattern.

As a caveat for all considerations put forward below, it should be kept in mind that the sample size in the current study is limited due to the general scarcity of time series in museum collections (even for these generally well-documented Japanese macaque populations).

### Differences in skull morphology among human impact groups

A recent study has shown mandibular shape differences but no size differences in Japanese macaques as a result of captivity (Kamaluddin et al. 2019). Similarly, the current analyses showed comparable skull shape differences among the human impact groups (wild, wild-provisioned, and captive) of Japanese macaques, in both allometry-adjusted (i.e., corrected for size) and non-allometry-adjusted data (Fig. 3). In particular, rostrum length appeared to discern the different human impact groups to some degree, with the captive group exhibiting a trend towards longer rostra compared to their wild conspecifics (Fig. 3). These differences among the human impact groups resemble the scenario of directional selection, with a trait changing away from the original mean and as a function of the number of generations in captivity (McPhee 2004). Reasons for such directional changes might be manifold and are outlined in the following paragraphs.

The feeding behaviour of the Japanese macaques has been studied in detail and varies with geographical area and captivity status, as well as with age and rank of the individual macaques (e.g., Tsuji 2010; Jaman and Huffman 2011). The natural food sources, which are variable with season, in wild populations (e.g., Kinkazan island) include leaves, flowers, fruits, buds, bark, fungi, seaweed, animal materials (fish, reptiles, insects, spiders, shellfish, fish), and soil (e.g., Watanabe 1989; Go 2010; Tsuji 2010). In the wild-provisioned Koshima population, the feeding regime has changed over time (Mori 1979): until 1963, only small amounts of food (mainly sweet potatoes and wheat) were provided on a low-frequency basis (much less than once a week); from 1964 onwards, intensive artificial feeding was conducted (soy beans). Besides the provisioned food sources, the Koshima population still uses natural foods (Go 2010). In the wild-provisioned Arashiyama population (i.e., the source population of the captive individuals of the Arashiyama population at KUPRI investigated here), the macaques consume both natural and provisioned food (Huffman and MacIntosh 2012). It has been observed that the macaques in the park consume about one third natural foods and two thirds provisioned food, the latter including wheat, soybeans, peanuts, chestnuts, and various fruits (e.g., apples) and vegetables (e.g., potatoes) (Wakibara et al. 2001; Huffman and MacIntosh 2012). Provisioned foods have been found to be higher in protein and carbohydrates compared to natural food (Wakibara et al. 2001). In a feeding experiment conducted with the captive Ueno Zoo Japanese macaque population, it was reported that the macaques receive different fruits (citrus, tomatoes), grasses, rice, different seeds (sunflower, wheat, hemp), a mix of branches and leaves of bamboo and other woody plants, and monkey chow, which is a commercial product designed to meet basic dietary requirements (Aoki et al. 2015). In studies on feeding behaviour in the Japanese macaques featuring the captive Takahama population housed at KUPRI, it has been described that the macaques receive commercial monkey chow supplemented with sweet potato several times a week (Takahashi et al. 2006). These studies suggest that the amount of natural food (e.g. leaves, fruits) appears to increase on average from captive to wild-provisioned to wild. Further, it might be speculated that the wild-provisioned populations experience the most favourable energy and nutrient intake because they can consume both natural and provisioned foods, whereas both in the wild and in captivity, energy and nutritional supply might be at least at times suboptimal due to seasonal variation in the wild and relatively uniform diet in captivity. The detailed feeding regime and its stability over time of the populations in the present work is unknown, and the age and rank of the individuals could not be considered. However, according to these considerations, body size and hence cranial and mandibular morphology (allometry) might vary due to variable feeding habits in the different populations studied here. On the other hand, a lack of mandibular size differences in a greater number of captive and wild Japanese macaques suggests similarity of feeding habits among the groups (Kamaluddin et al. 2019). Parasites influence body weight and life history variables.

In addition to nutritional influences, the mechanical properties of the diet and functional demands of feeding habits might play a role in the plastic response of the cranial and mandibular morphology and associated masticatory muscles (Groves 1966; Herring and Lakars 1981; Lieberman et al. 2004; O'Regan and Kitchener 2005; Paschetta et al. 2010; Hartstone-Rose et al. 2014; Cornette et al. 2015; Fabre et al. 2018; Siciliano-Martina et al. 2021). Specifically for primates, it has been found that squirrel monkeys (*Saimiri sciureus*) fed a soft diet exhibit a narrower muzzle than specimens which have been fed a harder diet (Corruccini and Beecher 1982). In humans, it has been suggested that the rapid and marked modifications of the physical and cultural environments since the agricultural and industrial revolution have led to smaller jaws (Kahn et al. 2020). Therefore, dietary and other mechanical differences between captive and wild populations, especially structurally unnatural food, and related variation in mechanical loading in captivity compared to the wild, might also contribute to differences in skull morphology between human impact groups. Parasite load has been reported in Japanese macaques (Mori 1979), but I am not aware of any studies documenting variable parasite load in captive versus wild or wild-provisioned macaques.

Further, hormonal differences between groups in captivity and in the wild might contribute to body size and skull shape differences among the human impact groups in this work. Specifically, it has been suggested that prolonged and elevated testosterone levels in captive social primates due to more extensive exposure to other males lead to more pronounced male features during ontogeny (Singleton 2012; Kamaluddin et al. 2019). Whether the same holds true for females, which are usually exposed to other conspecifics of their own sex in the wild, is unlikely but remains to be investigated (Singleton 2012). Alternatively, increased stress levels during growth are known to lead to psychosocial dwarfism via a reduced blood growth hormone level (Green et al. 1984). However, such considerations concerning the populations studied here remain highly speculative.

Besides the plastic responses to different captivity regimes as described above, the differences which were observed between the human impact groups might be the result of specific adaptations to a particular environment, including the diet. After all, the studied populations or the source populations of the captive groups have been distributed across Japan (Fig. 1). Ecomorphological variation has been studied relatively well in Japanese macaques (e.g., Nakagawa et al. 2010). Specifically, morphological and body size changes might be based on the insular habitat of the Kinkazan and Koshima populations, which has been suggested to lead to a distinct morphology (Hamada and Yamamoto 2010; Buck et al. 2018). Alternatively, Bergman’s and Allen’s rule, with populations from higher latitudes and a colder climate (e.g., Kinkazan) being larger than southern ones (e.g., Koshima), might influence skull proportions as well (Iwamoto 1971; Hamada et al. 1996; Hamada and Yamamoto 2010; Buck et al. 2018). A similar gradient has been suggested from the centre of Japan to the periphery on the basis of gene flow data (Hamada and Yamamoto 2010). Further, dietary differences between populations of these different climate zones might explain differences in molar size (Hamada and Yamamoto 2010; Asahara and Yuichiro 2017). Such body size and morphological variation might influence the variation which was observed among the human impact groups investigated here, although no such evidence was found in a large sample of captive and wild Japanese macaques regarding mandibular shape and size (Kamaluddin et al. 2019). For example, the wild Kinkazan population in the cold north might be larger and with increased molar size relative to the smaller-bodied wild-provisioned Koshima population in the south, which likely has implications for skull shape. On the other hand, both of these populations might exhibit similarities in skull shape due to their insular environment. Such multivariate geographical impacts were impossible to discern here given the limited data set. However, the two wild/wild-provisioned source populations of the most closely studied captive populations here (Arashiyama and Takahama) are geographically close, with their captive descendants even housed at the same facility (KUPRI, Inuyama, Fig. 1) under likely similar environmental conditions. This allows for better control of captivity effects (see next chapter). However, due to the lack of genetic data, it cannot be distinguished in the current study whether the observed inter-population morphological differences are caused by human impact or reflect inter-population genetic differences.

Lastly, the ‘domestication syndrome’ hypothesis has been prominent in the attempt to explain similar morphological changes occurring in otherwise unrelated domesticated populations (Trut 1999; Wilkins et al. 2014), although this hypothesis is disputed (e.g., Sánchez-Villagra et al. 2016; Lord et al. 2019). The typical characteristics of the domestication syndrome include smaller brain and facial size, for example, and these changes have been linked, amongst other factors, to selection for tameness and related developmental processes (e.g., Wilkins et al. 2014). It has been suggested that a similar process might inadvertently be occurring in captivity, due to the (unconscious) selection of individuals that are behaviourally compatible with the captive environment (O'Regan and Kitchener 2005). However, a study on captive wild boar and domestic pigs suggests different patterns of morphological change in captivity and domestication, respectively (Neaux et al. 2020). In the current study, no evidence for the domestication syndrome was found in the studied macaque populations. On the contrary, rostrum size seemed to be greater in at least some captive populations. One contributing factor here might be that even the wild (Kinkazan) populations are at least partly habituated to humans (Agetsuma and Nakagawa 1998), thus already advanced to a certain degree on the ‘domestication continuum’ (Vigne 2011; Zeder 2012, Table 1) and potentially showing characteristics of the domestication syndrome (Trut 1999; Geiger et al. 2018).

### Differences in skull morphology across the first generations in captivity

Apart from these considerations concerning potential directed genetic or plastic changes as an overall pattern describing morphological differences among the human impact groups (Fig. 3), this pattern did not seem to hold when the single captive populations were examined in detail (Table 4; Fig. 4). Changes over time in the two study populations (Arashiyama and Takahama) concerned body size reductions as well as body size-independent changes in cranial and mandibular shape (Fig. 4, Table 4). These shape changes, however, were not similar between the two populations. In particular, different skull dimensions were found to change throughout the first generations in captivity in the Arashiyama and the Takahama populations (Table 4, Fig. 4). This is despite the fact that the captive generations of both populations have been kept in the same institution (KUPRI, Inuyama; Fig. 1) and therefore probably under similar conditions. Further, the only skull measurement that has been found to change in both the Arashiyama and the Takahama populations, M1 breadth, was changing in different directions in both populations (Fig. 4). These results suggest that genetic drift due to small founder populations might shape cranial and mandibular morphological diversity in these populations (McPhee 2004). McPhee (2004) found that, although morphological disparity tended to increase with the number of generations in captivity in oldfield mice (*Peromyscus polionotus subgriseus*), the morphological changes were not uniform among the captive study populations, i.e., different changes occurred in different study populations, and not cumulative, i.e., changes in one generation might be reversed in the next. She interpreted this pattern as a result of relaxed selective pressures in captivity coupled with founder effects (McPhee 2004).

Body size changes, especially body size increase, are frequently reported in captive primates and linked to a better, more constant nutrition and medical care in captivity versus in the wild (Kimura and Hamada 1996; O'Regan and Kitchener 2005; Turner et al. 2016). However, morphological changes and body size changes due to captivity are not uniform. For example, comparisons of body dimensions and weight in wild-provisioned and captive rhesus macaques (*Macaca mulatta*) on Cayo Santiago showed that captive individuals were generally smaller at any given age compared to their wild-provisioned counterparts (Gore 1993). These differences have been hypothesised to be related to a diet more limited in certain nutrients (apparently not energy and proteins) in the captive group, and/or on founder effects due to relatively small-bodied founder females in the captive population (Gore 1993). The results reported here, which are showing a consistent body size decrease during the first generations in captivity in the Arashiyama and the Takahama populations (Table 4; Fig. 4), are in accordance with these findings on body size decrease in captive rhesus macaques, and underlying mechanisms might therefore be similar.

Interestingly, this body size decrease under high human impact is parallel to what is often reported in early domestication stages in many domestic animals (Tchernov and Horwitz 1991). A decrease in body size in domestication has been suggested to be the result of both plastic and adaptive responses, such as deliberate or unconscious selection for the trait and/or for higher reproductive rates. Further, it has been suggested that relaxed natural selection in a more unpredictable environment with higher intraspecific competition in high-density populations leads to increased fitness of smaller, early-reproducing individuals (Tchernov and Horwitz 1991). The latter might also be the case in the captive Arashiyama and Takahama populations at KUPRI investigated here.

### Rates of change in cranial and mandibular morphology

Changes in skull morphology over time could only be observed in the studied captive populations, whereas no evidence for such a change was discernible in the wild and wild-provisioned populations (Table 3). These results appear to support the hypothesis that skull dimension changes occur at a faster rate if human impact is high. However, although investigated time spans were similar among the populations, these results might also be influenced by differences in sample size (largest for the captive populations; Fig. 1; Table 3). The focus of the discussion of results will therefore be on the comparison of the investigated rates of change with comparable literature data.

As the changes observed here concern variation within one generation only, the estimated rates of change in Haldanes were compared to step rates from the literature. Step rates give the standard deviation per generation (i.e., Haldanes) on a timescale of one generation. In an extensive review, Gingerich (2019) estimated step rates in different plant and animal species in experimental selection and field settings, reflecting different intensities of animal–human interactions including wild, commensal, and captive. These comparisons resulted in median step rate estimates of 0.33 and 0.15 standard deviations per generation in experimental selection and field studies, respectively, wherein the range of step rates has been found to be similar between the two settings (Gingerich 2019). The average step rate found in the current sample for the Arashiyama and the Takahama populations (0.891 standard deviations per generation) is markedly higher than the literature samples, including the experimental selection and the field studies in an anthropogenic context, which—in a traditional view—would have been supposed to be represented by relatively high step rates. This result highlights the great selection pressure or great influence of genetic drift or phenotypic plasticity on the captive populations investigated here and corroborates the traditional view that evolution is particularly fast under high human impact. However, on the other hand, the range of step rates reported for field studies in a natural context, which according to the traditional view would contain the lowest step rates due to low human impact, also contains step rates much greater than the ones reported in the current Japanese macaque sample (Gingerich 2019).

Previous studies have suggested that rates of change are faster in human-dominated populations relative to those under less human impact (Hendry et al. 2008). However, studies on domestication and breed formation show that this is not necessarily the case (Purugganan and Fuller 2011; Geiger et al. 2018). In sum, and together with the current study, this evidence further crystallises the notion that rates of change in the wild, without or with only limited human impact, can be faster than previously expected, although rates of change in captivity might still be exceptionally fast.

## Conclusions

These data, despite caveats concerning limited sample size, possible influence of age, and lack of generational data in some populations, provide a rare opportunity to study morphological changes over time and related to variable human impact. This study contributes to the growing evidence that there is no uniform change in cranial and mandibular shape along the wild-domestic gradient (e.g., the ‘domestication syndrome’; Table 1) and that the rate of change may be fast, but not necessarily faster, if human influence on populations increases.

## Supplementary Information

Below is the link to the electronic supplementary material.

Supplementary file1 (XLSX 32 KB)

Supplementary file2 (PDF 356 KB)
