# Supplementary material for: Pattern and pace of morphological change due to variable human impact: the case of Japanese macaques
Source: Primates. 2021 Aug 17;62(6):955–70. doi: 10.1007/s10329-021-00933-7 (PMC8526449; doi:10.1007/s10329-021-00933-7)
Supplement: Supplementary file 2 — Supplementary file2 (PDF 356 KB) [file 10329_2021_933_MOESM2_ESM.pdf]

Electronic supplementary material

Pattern and pace of morphological change due to variable human impact – the case of Japanese macaques

Journal: Primates

Madeleine Geiger[1]*

[1] Palaeontological Institute and Museum, University of Zurich, Karl-Schmid-Strasse 4, CH-8006 Zürich, Switzerland

* Corresponding author: madeleine.geiger@uzh.ch

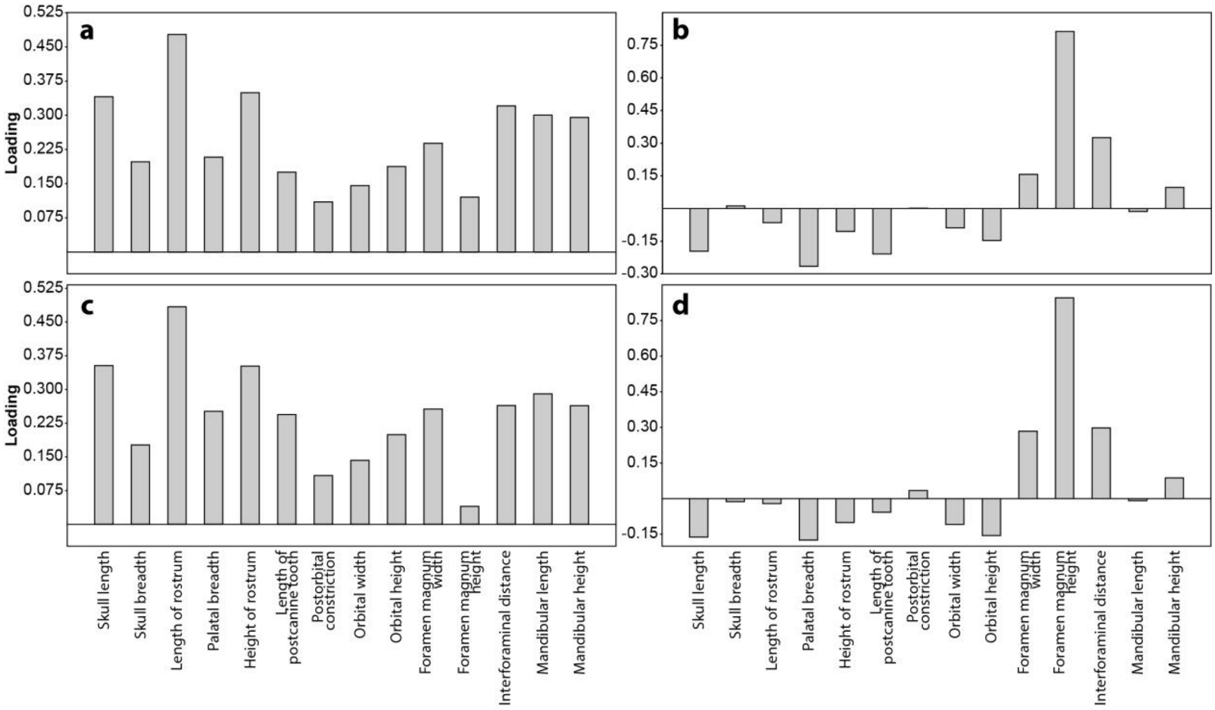

Fig. S1 Principal component (PC) coefficients showing loading of measurements on PC's. Non-allomety-adjusted principal component analysis of PC1 (a) and PC2 (b); allometry-adjusted principal component analysis of principal component 1 (PC1, c) and PC2 (d)
